# Sleep quality and BMI in pregnancy– a prospective cohort study

**DOI:** 10.1186/s12884-022-04414-7

**Published:** 2022-01-27

**Authors:** Yafang Tang, Fei Dai, Nurul Syaza Razali, Shephali Tagore, Bernard S. M. Chern, Kok Hian Tan

**Affiliations:** 1 Department of Obstetrics and Gynecology, KK Women’s and Children’s Hospital, Singapore, Singapore; 2 Department of Maternal Fetal Medicine, KK Women’s and Children’s Hospital, 100 Bukit Timah Road, Singapore, 229899 Singapore; 3 Department of Minimally Invasive Surgery, KK Women’s and Children’s Hospital, Singapore, Singapore

**Keywords:** Sleep, BMI, Pregnancy

## Abstract

**Background:**

Pregnancy associated sleep disturbances is a common pregnancy-related complication which can lead to significant maternal distress and adverse pregnancy outcomes. Sleep quality can be affected by multiple factors and obesity has been recognized as one of them. Various previous studies have demonstrated poorer sleep quality during pregnancy. However, most studies included assessment at only one point of pregnancy. This prospective cohort study aimed to better evaluate the effect of pregnancy on the quality of sleep throughout the antenatal period and how BMI affects antenatal sleep.

**Methods:**

A total of 926 women were recruited before 14 weeks of gestation and followed throughout pregnancy. The Pittsburgh Sleep Quality Index questionnaire (PSQI) was employed to assess sleep quality in 4 antenatal visits throughout pregnancy. Their weight was also recorded at each visit.

**Results:**

The PSQI global score was higher towards the later part of pregnancy (6.4 to 8.0, *p* < 0.001) and highest at the 4th visit. Sleep latency was longer as pregnancy progressed (18.5 mins to 23.2 mins, *p* = 0.001). Sleep duration became shorter over time and was the shortest at the 4th visit (7.1 h to 6.5 h, *p* < 0.001). Sleep efficiency was the lowest at the 4th visit (85.2 to 81.6%, *p* < 0.001). The same trend was observed for subjects in different BMI groups throughput pregnancy. PSQI score increased and sleep duration decreased as BMI increased. The effect of increasing BMI on PSQI and sleep duration was only observed in the higher BMI groups (> 25 kg/m^2^).

**Conclusions:**

Our study showed that sleep quality gradually declined throughout pregnancy for all BMI groups. Higher BMI was associated with poorer sleep as represented by PSQI score and sleep duration, particularly in the overweight and obese subgroups.

**Supplementary Information:**

The online version contains supplementary material available at 10.1186/s12884-022-04414-7.

## Background

Sleep disturbance has long been recognized as one of the common obstetric complications [1, 2]. Previous studies have shown sleep disturbances in all trimester throughout pregnancy [3, 4]. Shorter sleep duration, poorer sleep efficiency, insomnia and overall poor sleep quality characterize the sleep of antenatal women, especially in late pregnancy [5–8]. A review by Chang et al. reported mean sleep duration of 6.8 to 7.8 h per night before pregnancy, slightly increasing during the 1st trimester, then decreasing during the 2nd and 3rd trimesters [9]. Risk factors for poor sleep in pregnancy include poor pre-pregnancy sleep, maternal emotional disturbances (depression, anxiety, stress), body image, obesity, more weight gain during pregnancy, physiological changes during pregnancy such as decreased tone of esophageal sphincter, hormonal changes, increased uterine size and increased micturition [10–14]. Poor sleep during pregnancy can cause adverse health concerns for the pregnant women [15, 16] and can lead to potential adverse outcomes of pregnancy such as intrauterine growth restriction and preterm birth [17–19].

Most previous studies have focused on one point of time in pregnancy or were cross sectional studies involving multiple stages of pregnancy [1, 5, 7]. A prospective study by Plancoulaine et al., one of the few cohort studies, showed gradual decline in sleep duration as pregnancy progresses [20]. Our prospective study aimed to assess the change of sleep quality in detail (overall sleep quality, sleep duration, sleep efficiency and sleep latency) as pregnancy progresses. Subjects were followed from the first antenatal visit throughout the entire pregnancy, and sleep quality was assessed thoroughly at 4 visits. As obesity is increasingly prevalent globally and associated with sleep disorders such as sleep apnea and cause sleep disturbances in both non-pregnant and pregnant population [21–23], it is interesting to explore sleep quality in women in different body mass index (BMI) groups. One large cohort study involving 2366 pregnant women by Guinhouya BC et al. showed overall sleep quality was worst in 3rd trimester and obese women exhibit a greater PSQI score than women with normal weight [24]. However, large scale prospective cohort studies are scarce. Our study also aimed to provide a comprehensive comparison on various aspects of sleep through pregnancy among women in different BMI categories.

## Methods

The present study was part of the Neonatal and Obstetric Risk Assessment (NORA) study, a large prospective cohort study. Women with singleton pregnancies attending their first prenatal visit at KK Women’s and Children’s Hospital, Singapore, between September 1, 2010, and August 31, 2014, were invited to participate. All women with viable singleton pregnancies (confirmed by ultrasonography) and at less than 14 weeks of gestation were potentially eligible. Patients with chronic medical conditions such as renal disease and systemic lupus erythematosus, pregnancies complicated by aneuploidy or fetal anomalies, and pregnancies ending in termination, spontaneous abortion, or fetal death before 24 weeks of gestation were excluded. Gestational age was determined by fetal crown–rump length at the first prenatal ultrasonography examination. Enrolment details were described elsewhere [25]. Briefly, of the 3271 women identified, 1013 women consented to the study and 934 women completed all follow up visits (92%). Nine hundred twenty-six women had complete delivery record (96%). At recruitment, a detailed interview was carried out by clinical research coordinators while a dating ultrasound scan and routine antenatal blood collection were done by sonographers and nurses respectively. At subsequent follow up visits, clinical and laboratory data were collected at 18 to 22 weeks, 28 to 32 weeks and 34 weeks and above. Following delivery, detailed information on pregnancy complications, labor, delivery and neonatal outcomes was collected. The present study mainly focused on patient demographics, sleep quality throughout pregnancy and BMI.

Information on socio-demographic and life style characteristic was collected during the 1st antenatal visit and updated during each subsequent visit when applicable. Included were age, BMI, race, parity, marital status, education level, occupation and monthly income. For assessment of effect of BMI on sleep quality, subjects were grouped into underweight (< 18.5 kg/m^2^), healthy weight (18.5 - < 25 kg/m^2^), overweight (25 - < 30 kg/m^2^) and obese (30 kg/m^2^ and above) based on the BMI in the first visit.

Four study visits at the different gestational ages of pregnancy were conducted in the NORA Study (1st visit at 11-14 weeks, 2nd visit at 18-22 weeks, 3rd visit at 28-32 weeks and 4th visit after 34 weeks of gestation). During each visit, sleep quality was assessed via the Pittsburgh Sleep Quality Index (PSQI), which is a widely used and well-validated 19-item self-report questionnaire that measures sleep quality in adults [26]. It provides a global score ranging from no sleep difficulty to severe difficulties. Higher PSQI score indicates poorer sleep. Four main questions were asked in the questionnaire: (1) “During the past month, what time have you usually gone to bed at night”; (2) How long (in minutes) has it usually taken you to fall asleep each night”; (3) What time have you usually got up in the morning”; (4) How many hours of actual sleep did you get at night, which may be different from the number of hours you spent in bed”. Sleep duration is defined as question 4. Sleep latency is defined as question 2. Sleep efficiency is defined as sleep duration / hours in bed. Questions on whether there is difficulty in falling asleep, night awakening, bathroom use at night were also included in the questionnaire. PSQI score was calculated after adding the scores for each question.

Maternal demographics such as age and BMI were considered as continuous variables and were described as mean ± standard deviation. Other factors including race, education, marital status, occupation, income and parity were considered as categorical variables and were described as the sample number and frequency of each category. The mean PSQI, latency, duration and efficiency of sleep for each visit were described as mean ± standard deviation. Repeated measure ANOVA was employed to establish any significance difference in BMI and sleep quality throughout the four antenatal visits. Analysis was adjusted for age and SBP (continuous variables). Statistical significance was defined as *p* < 0.05. Mixed model analysis was employed to study the change in sleep quality at different stages of pregnancy and was performed with scaled identity as the repeated covariance type. Analysis was adjusted for age, race, education, coffee drinking, smoking, exercise, SBP and parity as potential confounders. Statistical significance was defined as *p* < 0.1 due to model complexity. Statistical analysis of the data was performed using SPSS 22.0 (IBM, Armonk, NY, USA).

## Results

The basic demographics of the study subjects were shown in Table 1. The average age of the study population was 30 years with an average BMI of 24.2 kg/m^2^ at the first visit. Majority of the subjects were of Chinese ethnicity (50.8%), followed by Malay which made up of 20.7% of the subjects. The remaining subjects were either Indians (10.8%) or others (11.4%). Most of the subjects have education levels of high school/junior college and above (98.4%). Their household income ranges from < 3500 (34.4%) to more than 8500 Singapore dollars a month, with most women working as white-collar workers (79.9%). Nulliparous women made up of 54.4% of the study subjects. 21% of women continued to drink coffee during pregnancy while only a small percentage of women continued to smoke (2.6%) or drink alcohol (1.2%). Only 8.4% women remained active in pregnancy and continued to exercise. Lastly, the average blood pressure at first visit was 108.6/65.8 mmHg.

**Table 1:** Maternal socio-demographics, life styles and blood pressure at first visit (11-14 weeks)

Maternal characteristics	*N* = 926	Mean ± SD / %
Age (years)	926	30.6 ± 5.0
BMI (kg/m^2^) (first visit)	921	24.2 ± 4.7
Race		
Chinese	470	50.8
Indian	100	10.8
Malay	250	27.0
Other	106	11.4
Missing	0	0
Education		
Secondary school or under	12	1.3
High school	209	22.6
Junior college	367	39.6
University or above	335	36.2
Missing	3	0.3
Marital status		
Married	871	94.1
Single/Divorced/Widowed	55	5.9
Missing	0	0
Occupation		
White-collar worker	740	79.9
Blue-collar worker	176	19.0
Unemployment	10	1.1
Missing	0	0
Total monthly household income (S$)		
< 3500	319	34.4
3500-5500	280	30.2
5501-8500	203	21.9
> 8500	122	13.2
Missing	2	0.2
Coffee drinking (yes, %)	194	21.0
Smoking (yes, %)	24	2.6
Drinking alcohol (yes, %)	11	1.2
Exercise (yes, %)	78	8.4
SBP (mmHg)	922	108.6 ± 11.3
DBP (mmHg)	922	65.8 ± 8.3
Parity		
0 previous birth	498	54.4
1+ previous birth	418	45.6
Missing	0	0

Continuous variables are expressed in mean ± SD while categorical variables are expressed as %

Sleep quality throughout pregnancy was demonstrated in Table 2. Sleep efficiency was significantly different throughout pregnancy (*p* < 0.001) with the highest during the 1st visit (85.2%) and lowest at the 4th visit (81.6%). PSQI score varied significantly among the visits as well with the lowest during the 2nd visit (6.2) and highest during the 4th visit (8.0) (*p* < 0.001). Sleep latency was longer when pregnancy was more advanced with the longest latency of 23.2 mins at the 4th visit compared to 18.5 mins at the 1st visit (*p* < 0.001). Sleep duration gradually shortened as pregnancy progresses, from 7.1 h during the 1st visit to 6.5 h during the 4th visit (*p* < 0.001).

**Table 2:** BMI and sleep quality measurements throughout 4 antenatal visits

BMI group		11-14 weeks		18-22 weeks		28-32 weeks		> 34 weeks		F	P
	N	Mean	SD	Mean	SD	Mean	SD	Mean	SD		
**BMI (kg/m**^**2**^**)**	780	24.0	4.7	25.2	4.5	27.1	4.5	28.2	4.8	1914.5	< 0.001*
< 18.5	61	17.3	1.0	18.8	1.2	20.8	1.5	21.9	1.8	13.2	< 0.001*
18.5-24.9	443	21.8	1.7	23.0	1.8	25.1	2.1	26.3	3.1	1049.3	< 0.001*
25-29.9	192	27.3	1.5	28.3	1.7	30.0	2.0	31.1	2.2	294.6	< 0.001*
≥ 30	84	33.4	3.3	34.0	3.1	35.5	3.5	36.6	4.1	185.2	< 0.001*
**Sleep efficiency (%)**	758	85.2	14.3	85.1	14.8	83.3	15.0	81.6	16.5	8.8	< 0.001*
< 18.5	59	83.7	15.2	83.4	15.3	82.6	16.9	80.9	19.1	1.9	0.137
18.5-24.9	431	84.9	13.6	85.1	14.9	83.3	14.5	82.3	15.8	3.6	0.013*
25-29.9	186	84.9	15.4	85.2	14.3	82.6	15.7	80.6	17.5	0.5	0.679
≥ 30	82	88.3	14.5	86.2	15.5	85.0	14.6	80.8	16.0	6.8	< 0.001*
**PSQI Score**	749	6.4	3.0	6.2	3.1	7.1	3.3	8.0	3.3	74.1	< 0.001*
< 18.5	58	6.3	2.9	6.2	3.5	6.6	3.3	7.9	3.3	3.4	0.026*
18.5-24.9	428	6.5	3.1	6.2	3.0	7.1	3.4	7.9	3.2	20.7	< 0.001*
25-29.9	182	6.4	3.0	6.0	3.4	7.0	3.2	8.0	3.3	20.6	< 0.001*
≥ 30	81	6.5	3.2	6.5	3.2	7.6	3.1	8.4	3.4	18.5	< 0.001*
**Sleep latency (mins)**	747	18.5	17.0	18.4	16.8	21.0	19.0	23.2	20.4	12.0	< 0.001*
< 18.5	58	18.4	18.0	17.4	18.7	20.4	18.3	20.4	17.2	3.0	0.041*
18.5-24.9	429	18.1	15.8	18.1	16.0	21.0	19.6	22.8	19.7	4.1	0.006*
25-29.9	179	17.0	14.3	17.3	16.2	19.9	18.0	22.1	17.4	4.5	0.004*
≥ 30	81	24.2	25.0	22.9	20.0	24.0	18.0	29.4	29.3	1.2	0.324
**Sleep duration (hours)**	760	7.1	1.5	7.0	1.5	6.6	1.5	6.5	1.7	23.0	< 0.001*
< 18.5	60	7.3	1.6	6.9	1.5	6.9	1.7	6.5	1.8	0.1	0.971
18.5-24.9	432	7.0	1.5	7.0	1.5	6.6	1.5	6.5	1.7	9.0	< 0.001*
25-29.9	186	7.1	1.6	7.0	1.6	6.6	1.5	6.5	1.8	3.0	0.03*
≥ 30	82	7.1	1.8	7.0	1.5	6.6	1.6	6.4	1.6	5.3	0.001*

Repeated measure ANOVA analysis. Statistical significant is defined as *p* < 0.05

BMI gradually increased as pregnancy progressed as expected, from 24 kg/m^2^ in the first visit to 28.2 kg/m^2^ in the 4th visit (Table 2). At the first visit, majority of subjects were in the normal BMI group (443/780, 56.8%) with a significant percentage overweight (192/780, 24.6%) or obese (84/780, 10.8%). Sleep quality change in the 4 different BMI groups generally follow the same trend as that of the whole study population, with worsening sleep efficiency, higher PSQI scores, longer sleep latency and shorter sleep duration as pregnancy progressed (Table 2). Difference for some of the subgroups did not achieve statistical significance such as PSQI score for the BMI < 18.5 kg/m^2^ group. It can partly be explained by type II error due to the small sample size (58) in that group.

Correlation between BMI and sleep throughout pregnancy was then explored further using mixed model analysis. Table 3 showed that PSQI score was higher with increasing BMI (*p* = 0.03) while sleep duration was shorter with increasing BMI (*p* = 0.08). Upon closer scrutiny, the subgroups with higher BMI (BMI 25-29.9 kg/m^2^ and ≥ 30 kg/m^2^) seemed to be the ones with their sleep quality significantly affected by increasing BMI. In the group of subjects with BMI between 25 and 29.9 kg/m^2^, sleep duration was significantly shorter when BMI increased (*P* = 0.005). For the subjects with BMI ≥30 kg/m^2^, PSQI score was significantly higher with increasing BMI (*p* = 0.015) and sleep duration was shorter (*p* = 0.056).

**Table 3:** Mixed model analysis of the correlation between BMI and sleep (log) throughout 4 antenatal visits

BMI (kg/m2)	Efficiency		PSQI score		Latency		Duration	
	β	P	β	P	β	P	β	P
< 18.5	−0.007	0.598	−0.019	0.472	0.072	0.088	−0.019	0.216
18.5-24.9	0.002	0.322	0.004	0.352	−0.002	0.785	0.000	0.944
25-29.9	−0.002	0.321	0.004	0.338	0.007	0.358	−0.007	0.005*
≥30	− 0.001	0.382	0.007	0.015*	0.004	0.497	− 0.003	0.056*
total	0.000	0.595	0.003	0.030*	0.003	0.149	−0.001	0.080*

Mixed model analysis. Statistical significance is defined as *p* < 0.1

## Discussion

Our study showed that sleep quality progressively worsens as pregnancy advances, with higher PSQI score, longer latency, shorter duration and poorer efficiency. Women in different BMI groups generally followed a similar trend while the correlation between higher BMI and worse sleep quality was more pronounced in the overweight and obese groups.

Sleep disturbances are widely recognized throughout pregnancy with more previous studies reporting sleep disturbances in later trimesters. A cohort study by Plancoulaine et al. showed that sleep duration decreased from 8.0 h in first trimester to 7.2 h in third trimester [19]. However, overall sleep quality and other aspects of sleep including sleep latency and efficiency were not assessed in that study. Another small cohort study published recently showed that sleep duration (8.1 in first trimester and 7.5 in third trimester) was not significantly different throughout pregnancy [27]. However, that study consisted of 176 women only and the non-significant result could be due to type II error. Guinhouya et al’s cohort study demonstrated best sleep quality in 2nd trimester and no significant difference in sleep duration throughout 3 trimesters [24]. Our study which studied comprehensively, overall sleep quality, sleep duration, sleep efficiency and sleep latency, showed that sleep disturbances are most severe in the last antenatal visit, consistent with most previous studies. The reason for the progression of sleep disturbances may partially be explained by the different mechanisms contributing to poor sleep in different stages of pregnancy. In the first trimester, potential contributing factors include urinary frequency, heartburn, hyperemesis [28, 29]. As pregnancy progresses to the second trimester, the effect of estrogen and progesterone may cause tissue edema and result in obstructive sleep apnea [30]. In the last trimester, the gravid uterus, urinary frequency, abdominal and back pain, gastroesophageal reflux play important roles in the worsening of sleep quality [31, 32]. Meanwhile, pre-pregnancy sleep disorders, mood disorders and emotional stress should not be underestimated [11].

Obesity has been recognized as a risk factors for poor sleep quality antenatally [33]. In a large-scale prospective study (*n* = 2366), Guinhouya et al. showed that pre-pregnancy high BMI correlates with poorer overall PSQI score [22]. Another study by Vézina-Im et al. showed no difference in sleep duration among women in different BMI groups [34]. Our study employed mixed model analysis and showed that overall PSQI score was poorer and sleep duration was shorter with increasing BMI. This correlation was mainly contributed by the overweight and obese groups. Obesity can potentially affect sleep by increasing risk of sleep apnea and other sleep disorders. Possible mechanisms include increased fat deposition around the upper airway causing obstruction of breathing and reducing the flow of oxygen [35]. Maintaining healthy body weight and BMI may potentially improve sleep in pregnant women.

Our study did not include the preconception and postpartum phase for comparison of sleep quality. C Hedman et al. reported that sleep duration was the shortest 3 months postpartum [36]. Future studies may consider including pre-pregnancy and postnatal sleep quality as it would better demonstrate the natural progression of sleep disturbances during pregnancy and postpartum period. Depression and anxiety can potentially affect sleep independent of pregnancy and were described to be possible factors associated with poorer sleep in a few previous studies [37, 38]. Previous study has also shown that obesity and depression tend to co-occur within individuals [39]. The relationship among obesity, mood disorders and sleep quality are complex. Our study did not include depression or anxiety in statistical analysis and it should be considered in future studies.. Information on shift work was not included in our study and it should be considered in future studies as sleep quality can potentially be affected significantly. Sleep quality was assessed purely with questionaries which was limited by potential subjective bias / recall error. Objective assessment of sleep quality such as polysomnography can be considered in future studies although it may not be cost effective. Our study is an observational study without any intervention on sleep being investigated. Interventions to improve sleep could be explored in future and offered to pregnant women with sleep disturbances to achieve the best maternal and fetal outcome. One interesting study reported significant improvement in sleep in terms of longer sleep duration and less sleep disruption with home-based cognitive-behavioral training (CBT) program during late pregnancy [40]. CBT was also explored in a study in obese women which showed improvement in insomnia symptoms [41]. A recently published systematic review which included studies on CBT, pharmacotherapy, acupuncture, mindfulness and herbal remedies, emphasized however that there is still lack of evidence for preventive and treatment options for sleep problems in pregnancy [42]. Future studies are still needed due to the paucity of data.

## Conclusion

In conclusion, this study is the first large prospective cohort study that evaluated detailed sleep quality throughout pregnancy. We showed that sleep disturbances progressively worsen with the advancement of pregnancy. Obesity is a potential factor that is associated with poorer sleep during pregnancy, particularly for the overweight and obese women. As good quality of sleep is associated with maternal well-being and good fetal outcome, it is important to advise pre-conception or antenatal patients to adopt healthy life style, maintain normal BMI and recognize sleep disturbances throughout the antenatal period and intervene appropriately to prevent adverse outcomes.

## Supplementary Information

**Additional file 1.**

## Data Availability

The datasets used and/or analysed during the current study are not publicly available due to limitations of ethical approval involving the patient data and anonymity, but are available from the corresponding author on reasonable request.

## Abbreviations

NORA: Neonatal and obstetric risk assessment
PSQI: Pittsburgh Sleep Quality Index
BMI: Body mass index
CBT: Cognitive-behavioral training
