# Supplementary material for: Sleep quality and BMI in pregnancy– a prospective cohort study
Source: BMC Pregnancy Childbirth. 2022 Jan 27;22:72. doi: 10.1186/s12884-022-04414-7 (PMC8793200; doi:10.1186/s12884-022-04414-7)
Supplement: Supplementary file 1 — Additional file 1. [file 12884_2022_4414_MOESM1_ESM.docx]

Questionnaire on lifestyle

1. Are you a coffee drinker before this pregnancy?

2. Did you consume any coffee during this pregnancy?

3. Did you smoke before this pregnancy?

4. Did you smoke during this pregnancy period?

5. Did you consume alcohol before this pregnancy?

6. Did you consume alcohol during this pregnancy?

7. Did you do any physical exercise before this pregnancy?

8. Did you do any physical exercise during this pregnancy?

9. Did you take any recreational drugs before this pregnancy?

10. Did you take any recreational drugs during this pregnancy?
